# 阿伐曲泊帕联合标准免疫抑制治疗伴肝损害的重型再生障碍性贫血6例

**DOI:** 10.3760/cma.j.issn.0253-2727.2022.11.012

**Published:** 2022-11

**Authors:** 建平 李, 文睿 杨, 园 李, 佑祯 熊, 蕾 叶, 慧慧 樊, 康 周, 洋 杨, 广新 彭, 馨 赵, 丽萍 井, 莉 张, 凤奎 张

**Affiliations:** 中国医学科学院血液病医院（中国医学科学院血液学研究所），实验血液学国家重点实验室，国家血液系统疾病临床医学研究中心，细胞生态海河实验室，天津 300020 State Key Laboratory of Experimental Hematology, National Clinical Research Center for Blood Diseases, Haihe Laboratory of Cell Ecosystem, Institute of Hematology & Blood Diseases Hospital, Chinese Academy of Medical Sciences & Peking Union Medical College, Tianjin 300020, China

再生障碍性贫血（AA）是由T淋巴细胞介导的、以骨髓造血组织为靶器官的自身免疫性疾病，免疫抑制治疗（IST）可使60％～70％的重型AA（SAA）患者获得血液学反应，无效患者被认为主要与残存造血细胞过少相关[1]。血小板生成素受体激动剂（TPO-RA）与造血干/祖细胞TPO受体作用，可明显增加AA患者骨髓多能造血祖细胞数量[2]，联合标准IST可显著提高初诊SAA患者血液学反应率和完全血液学反应率，缩短造血恢复时间，已被纳入SAA一线治疗选择[3]。

然而TPO-RA艾曲泊帕治疗后肝损害的报告多见。Townsley等[2]研究显示，艾曲泊帕治疗AA后18％的患者出现3～4级血清转氨酶和（或）胆红素升高；群体药代动力学研究显示，肝损害患者艾曲泊帕重复给药血浆浓度时间曲线下面积（AUC）升高111％～183％[4]；因此，原发免疫性血小板减少症（ITP）伴肝损害患者不推荐使用艾曲泊帕治疗。阿伐曲泊帕是与艾曲泊帕相似的一种新型小分子TPO-RA，可与人TPO受体的跨膜结构域相互作用，启动信号级联反应，诱导骨髓祖细胞增殖和分化。阿伐曲泊帕在慢性肝病相关血小板减少和成人慢性ITP（CITP）的研究中均取得了良好的治疗反应，不良反应较少，即使在慢性肝病患者中也显示出良好安全性，已被美国FDA批准用于上述疾病的临床治疗[5]。本研究中，我们尝试应用阿伐曲泊帕联合抗胸腺/淋巴细胞球蛋白（ATG/ALG）+环孢素A（CsA）治疗了6例伴肝损害的SAA患者，现报道如下。

## 病例与方法

1. 病例：以2021年3月至2021年10月于我院贫血诊疗中心住院接受阿伐曲泊帕联合IST且合并肝损害的初治SAA患者为研究对象。AA诊断符合1987年国际粒细胞减少和AA诊断标准[6]，严重程度分型参照Camitta标准[7]，ANC<0.2×10^9^/L的SAA诊断为极重型AA（VSAA）。所有患者均行染色体核型分析、外周血细胞流式细胞术CD55、CD59检查排除低增生性骨髓增生异常综合征（MDS）及阵发性睡眠性血红蛋白尿症（PNH）。肝功能损害分级标准参考常见不良反应术语评定标准（CTCAE）5.0版。

2. 治疗方案：（1）兔抗人胸腺细胞免疫球蛋白（rATG）3.0 mg·kg^−1^·d^−1^或猪抗人胸腺细胞免疫球蛋白（pALG）20 mg·kg^−1^·d^−1^静脉输注，连用5 d；同时给予泼尼松1 mg·kg^−1^·d^−1^预防即刻不良反应和血清病反应，2周后泼尼松开始减量，3周停药。出现血清病反应者临时给予氢化可的松100 mg/d静脉滴注。（2）患者疾病诊断明确后即开始CsA口服，以3 mg·kg^−1^·d^−1^分两次口服起始，一周后检测血药浓度，调整CsA全血谷浓度为200～400 µg/L。口服CsA至少6个月，当达到最佳血液学反应并血液学参数稳定至少3个月后开始缓慢减量，每3个月约减量1 mg·kg^−1^·d^−1^；口服CsA治疗共2～3年。（3）阿伐曲泊帕40 mg/d，自ATG/ALG应用第1天开始，至IST后6个月停药；PLT>100×10^9^/L进入剂量调整流程：PLT快速升高且>400×10^9^/L时停用阿伐曲泊帕，直至PLT≤100×10^9^/L后再继续阿伐曲泊帕20 mg/d口服；PLT>200×10^9^/L持续2周以上或>100×10^9^/L持续2个月以上，阿伐曲泊帕减量至20 mg/d，若PLT>200×10^9^/L持续2周以上或>100×10^9^/L持续2个月以上，阿伐曲泊帕继续减量至20 mg隔日1次，若PLT>200×10^9^/L持续2周以上或>100×10^9^/L持续2个月以上则停药。（4）支持治疗：在粒细胞缺乏期及ATG/ALG治疗期间予千级层流病房保护性隔离；ANC<0.5×10^9^/L时采用G-CSF 300 µg/d皮下注射，HGB<70 g/L及PLT<20×10^9^/L时分别给予红细胞、血小板输注。

3. 疗效观察：治疗后3个月内死亡定义为早期死亡，纳入治疗相关不良反应及疗效评价分析。疗效判定参照文献[2]标准：脱离红细胞和血小板输注，HGB≥100 g/L、ANC≥1.0×10^9^/L且PLT≥100×10^9^/L判定为完全治疗反应（CR）；脱离红细胞和血小板输注，ANC>0.5×10^9^/L、HGB≥70 g/L且PLT≥20×10^9^/L，而未达CR标准者判定为部分治疗反应（PR）；未达PR标准患者定义为无治疗反应（NR）。生存时间定义为IST开始至患者死亡或随访终点。所有患者分别于IST后3、6个月进行随访，主要随访指标包括外周血和骨髓细胞形态学、PNH克隆、脏器功能等以评估血液学反应和不良反应。

## 结果

1. 患者基本特征：6例初诊SAA/VSAA患者，VSAA 5例，SAA 1例；男5例，女1例，中位年龄37.5（12～55）岁；5例为原发性SAA，1例为肝炎相关SAA。应用rATG治疗 1例，pALG 治疗5例。IST前中位HGB 63（55～76）g/L，ANC 0.15（0.01～0.39）×10^9^/L，淋巴细胞绝对计数（ALC）0.88（0.30～2.03）×10^9^/L，网织红细胞绝对计数（ARC）8.1（2.7～45.3）×10^9^/L，PLT 5（2～8）×10^9^/L；2例患者伴有 PNH克隆，分别为4.7％和58.2％，均无临床溶血表现和生化检查溶血证据，余4例未检出PNH克隆。染色体核型检测均为正常核型。SAA诊断至开始IST中位时间为23（14～45）d。

2. 患者肝损害情况：例1～例4为发病后院外短暂治疗出现药物性肝损害；例5为慢性活动性乙型肝炎病毒感染致肝损害，HBV-DNA定量为7 000拷贝数/ml；例6入院前3个月罹患不明原因急性黄疸性肝炎，重度肝损害，为肝炎相关性SAA。参照CTCAE（2017年5.0版）标准，4例药物性肝损害患者IST开始时合并肝功能异常，均有胆红素升高，1例1级、1例2级、2例3级；转氨酶升高2例，均为3级；例5就诊时合并2级转氨酶和胆红素升高，经恩替卡韦抗病毒及保肝治疗1个月后于IST时开始HBV-DNA定量<1 000拷贝数/ml，转氨酶和胆红素恢复正常；例6 IST前3个月合并4级胆红素和转氨酶升高，经保肝治疗后好转，至IST开始时转氨酶和胆红素恢复正常（表1）。

**表1:** 6例SAA/VSAA患者阿伐曲泊帕治疗前后肝损害情况

例号	年龄（岁）	性别	诊断	肝损害原因	胆红素异常分级		转氨酶异常分级	
					基线	治疗后3个月	基线	治疗后3个月
1	51	男	VSAA	药物性	2	0	3	0
2	55	女	VSAA	药物性	1	0	0	0
3	30	男	VSAA	药物性	3	1	0	0
4	25	男	VSAA	药物性	3	0	3	0
5^a^	45	男	SAA	乙型肝炎病毒	2→0	0	2→0	0
6^b^	12	男	VSAA	不明原因	4→0	0	4→0	0

注 SAA：重型再生障碍性贫血；VSAA：极重型再生障碍性贫血；^a^慢性活动性乙型肝炎病毒感染患者，经恩替卡韦抗病毒及保肝治疗于免疫抑制治疗（IST）开始时肝功能恢复正常；^b^肝炎相关性SAA患者，IST前合并4级胆红素和转氨酶升高，经保肝治疗后好转至IST开始时肝功能检测恢复正常

3. 血液学反应：应用阿伐曲泊帕联合IST后，5例患者外周血参数较快上升，治疗2个月获得血液学反应，均为PR；至IST后3个月2例获CR，3例PR，1例NR，总体血液学反应率为83.3％，CR率为33.3％。IST后3个月获得血液学反应的5例患者血小板较快脱离输注，PLT>20×10^9^/L时间为治疗后21（14～28）d；ARC>40×10^9^/L时间为28（0～35）d，脱离输血的中位时间为35（28～56）d。IST后3个月进行骨髓穿刺评估造血恢复状况，6例患者均较治疗前明显好转，造血面积显著增加。至随访结束，6例患者全部获得血液学反应，2例CR，4例PR，总体血液学反应率为100.0％，CR率达33.3％。

4. 肝功能监测：4例基线时合并药物性肝功能损害患者，在阿伐曲泊帕治疗过程中原有肝功能异常无进一步加重。例1伴有2级胆红素升高，治疗后20 d恢复正常；3级转氨酶升高，治疗后28 d恢复正常。例2伴1级胆红素升高，治疗后14 d恢复正常。例3伴有3级胆红素升高，治疗后28 d降为2级，48 d降为1级。例4伴有3级胆红素升高和3级转氨酶升高，前者治疗后7 d降至1级，21 d恢复正常；后者35 d恢复正常。例5、例6基线时肝功能检测正常，治疗过程无新发肝功能异常表现。

5. 不良反应：例1合并脑出血，治疗后好转，考虑为SAA致血小板减少有关，与阿伐曲泊帕治疗无关；例5在初始服用阿伐曲泊帕40 mg/d时出现头痛症状，减量至20 mg/d口服后症状缓解，评估为阿伐曲泊帕治疗相关不良反应。一周后重新加量至40 mg/d口服，患者未再出现前述症状。6例患者无早期死亡，无原肝功能异常加重和新发肝功异常，无血栓并发症和消化道不良反应。合并慢性活动性乙型肝炎患者治疗过程中HBV-DNA定量持续<1 000拷贝数/ml，无肝炎病毒再活化。

6. PNH克隆转归：2例治疗前伴有PNH克隆患者，治疗后3个月均获得血液学反应，例2 PNH克隆由58.2％降至20.0％，无临床溶血表现和生化溶血证据。例6 PNH克隆消失，未再检出。

7. 生存及随访结果：6例患者均存活，无失访，中位随访127（98～180）d。至随访结束无患者出现细胞遗传学异常，无骨髓增生异常综合征/急性髓系白血病（MDS/AML）转化和骨髓纤维化发生。

## 讨论

与艾曲泊帕不同，阿伐曲泊帕的代谢主要是通过CYP3A和CYP2C9两种肝酶进行，不会导致胆红素水平升高；同时阿伐曲泊帕也没有金属离子螯合作用，与食物同时服用不影响吸收，有研究显示与食物一起服用可能吸收更好[8]。患者年龄（18～86岁）、体重（39～175 kg）、性别、种族、任何级别的肝功能损害和轻度至中度肾功能损害（肌酐清除率≥30 ml/min）对阿伐曲泊帕的药代动力学均无有临床意义的影响。阿伐曲泊帕已被FDA批准用于围手术期的慢性肝病（CLD）患者的血小板减少症和成人慢性ITP患者的治疗[9]。目前已有阿伐曲泊帕联合IST治疗肝炎相关SAA的个例报告，提示患者在获得血液学反应的同时肝功能逐渐好转[10]。我们报告阿伐曲泊帕联合IST治疗伴有肝损害SAA的6例患者全部获得血液学反应，并且原有肝功能损害呈恢复趋势，提示了其有效性和安全性。

艾曲泊帕不受体内干扰素-γ影响，可与人TPO受体的跨膜结构域相互作用，诱导骨髓祖细胞增殖和分化[11]。艾曲泊帕联合标准IST一线治疗SAA的研究显示，3个月总体血液学反应率为59％～80％，完全血液学反应率为22％～30％，脱离输血时间为32～51 d，相较于单纯IST治疗组均有显著提高，并且长期随访发现MDS/AML转化率没有升高，推荐艾曲泊帕联合IST作为一线不适合移植的SAA/VSAA患者的标准治疗[2],[12]。国内一项关于艾曲泊帕治疗AA的疗效与安全性的多中心调查研究显示，艾曲泊帕治疗可加快血液学反应的获得，改善反应质量[13]。目前尚没有关于阿伐曲泊帕联合IST治疗SAA疗效的临床研究。体外促进人脐带血CD34^+^细胞形成巨核细胞集落的研究以及在健康受试者的研究均显示，阿伐曲泊帕较艾曲泊帕有更强的增加PLT的作用[14]。阿伐曲泊帕可显著提升ITP患者PLT水平及维持疗效[15]–[16]。我们尝试阿伐曲泊帕治疗伴有肝损害的SAA患者，获得较好的血液学反应，3个月总体反应率为83.3％（5/6），CR率为33.3％（2/6）；3个月获得血液学反应的患者脱离输血中位时间为35 d；与艾曲泊帕联合IST治疗SAA的结果相当，提示阿伐曲泊帕联合IST治疗SAA亦可加快血液学反应的获得，改善反应质量。

阿伐曲泊帕的临床研究均没有发现药物相关肝功能异常[16]–[17]，不良反应发生率总体与安慰剂组相当，最常见不良反应为头痛[16]。鉴于阿伐曲泊帕用于慢性肝病患者更高的安全性，我们尝试应用了阿伐曲泊帕联合IST治疗伴有肝功能损害的SAA/VSAA患者。本研究共纳入6例患者，仅有1例患者服用阿伐曲泊帕40 mg/d后出现头痛症状可能与药物有关，减量至20 mg/d后症状缓解，并且维持一周后再次加量至40 mg/d未出现不适症状，无其他药物相关不良反应。肝功能监测结果显示在治疗过程中及治疗后均未出现原有肝功能损害加重，并且随时间延长肝功能逐渐好转，支持阿伐曲泊帕治疗伴有肝功能损害的SAA/VSAA安全性良好。

关于TPO受体激动剂对PNH克隆影响尚不清楚，已有报道艾曲泊帕联合IST治疗SAA并没有增加患者溶血性PNH转化率升高[13]。我们的研究中纳入2例伴有PNH克隆的患者，治疗后3个月均获得血液学反应，检测PNH克隆均呈减小趋势，1例患者消失，另1例较大克隆患者PNH克隆显著减小。结合艾曲泊帕相关研究提示TPO受体激动剂可能并不会促进PNH转化，但仍需更进一步研究以证实。

已有报道在艾曲泊帕停药后30％～50％的患者可能出现血小板下降，甚至病情反复[2]。我们的研究中无复发患者，在IST后3个月获得CR的2例患者都进入了阿伐曲泊帕减量流程，2例患者已经停药，无PLT下降至100×10^9^/L以下者。这可能与我们纳入的研究病例数较少、观察期较短或是我们的阿伐曲泊帕减量方案有关。

多数情况下，SAA患者血清TPO水平明显升高，NIH研究中提示血清TPO水平偏低的SAA患者在应用TPO受体激动剂联合IST后血液学反应率更高[2]。研究显示慢性肝病合并的血小板减少与血清TPO水平下降明显相关[18]。由于实验室条件限制，我们的研究并没有检测患者TPO水平；SAA患者合并肝功能损害时血清TPO水平变化尚不清楚，但我们的研究中6例合并肝损害的患者6个月全部获得血液学反应，提示这部分患者可能更需要联合TPO受体激动剂的治疗，而不受肝功能状况影响的阿伐曲泊帕似乎是更优选择。

我们报道了6例伴肝损害的SAA患者经阿伐曲泊帕联合IST后均获得血液学反应，较快脱离血小板输注，并且没有发生肝脏不良反应，初步提示其安全有效。由于我们的研究纳入病例数较少，随访周期短，缺乏对照组，阿伐曲泊帕联合标准IST的长期疗效和安全性仍需进一步研究。
